# An examination of the quinic acid utilization genes in *Aspergillus niger* reveals the involvement of 2 pH-dependent permeases

**DOI:** 10.1093/g3journal/jkaf199

**Published:** 2025-08-25

**Authors:** Michael Sgro, Ian D Reid, Mark Arentshorst, Arthur F J Ram, Adrian Tsang

**Affiliations:** Department of Biology, Concordia University, Montreal, Quebec H4B 1R6, Canada; Centre for Structural and Functional Genomics, Concordia University, Montreal, Quebec H4B 1R6, Canada; Centre for Structural and Functional Genomics, Concordia University, Montreal, Quebec H4B 1R6, Canada; Institute of Biology Leiden, Microbial Sciences, Leiden University, 2333 BE Leiden, South Holland, The Netherlands; Institute of Biology Leiden, Microbial Sciences, Leiden University, 2333 BE Leiden, South Holland, The Netherlands; Department of Biology, Concordia University, Montreal, Quebec H4B 1R6, Canada; Centre for Structural and Functional Genomics, Concordia University, Montreal, Quebec H4B 1R6, Canada

**Keywords:** *Aspergillus niger*, filamentous fungi, quinic acid catabolism, permease, transporter, gene knockout, transcriptomics

## Abstract

Many microorganisms are able to use plant-derived aromatic and cyclic compounds like the common plant secondary metabolite quinic acid as carbon and energy sources. In fungi, 3 enzymatic steps convert quinic acid into the common intermediate protocatechuic acid, which is then further converted into tricarboxylic acid cycle intermediates. The genes encoding these 3 enzymes are known to be part of a gene cluster in *Neurospora crassa* along with a permease, a gene of unknown function, and an activator-repressor module controlling expression of the cluster. This gene cluster is conserved in fungi and has also been studied in *Aspergillus nidulans*, where an additional gene of unknown function is included. Here, we studied these genes in the filamentous fungus *Aspergillus niger*, where the availability of high-quality, well-annotated genomes and efficient tools for genome-editing and global gene expression analysis could provide new insights into quinic acid utilization in fungi. Using homology and whole transcriptome sequencing, we identified the genes involved in quinic acid utilization. Knockout mutants of these genes were then created to observe the growth phenotype on quinic acid media. We showed that not all the genes involved in quinic acid utilization in *A. niger* are linked. In addition to the in-cluster permease gene, we identified a second, previously unknown off-cluster permease gene which was upregulated in the presence of quinic acid. These 2 permeases were determined to function optimally at different pH levels, with the in-cluster permease being more effective at pH 6.5 and the off-cluster permease more effective at pH 3.5.

## Introduction

Quinic acid is a common secondary metabolite in plants, comprising up to 10% of the weight of decaying leaf litter (Hawkins et al. 1993). As a precursor for products like chlorogenic acid, shikimic acid, and protocatechuic acid, quinic acid is involved in many plant processes including lignification, defense, and the synthesis of aromatic amino acids and secondary metabolites (Maher et al. 1994; Hoffmann et al. 2004). Industrially, quinic acid can be used to produce hydroxycinnamoyl-quinic acids, an increasingly valuable group of compounds used in pharmaceuticals, cosmetics, or as food additives (Valanciene and Malys 2022).

**Fig. 1.**
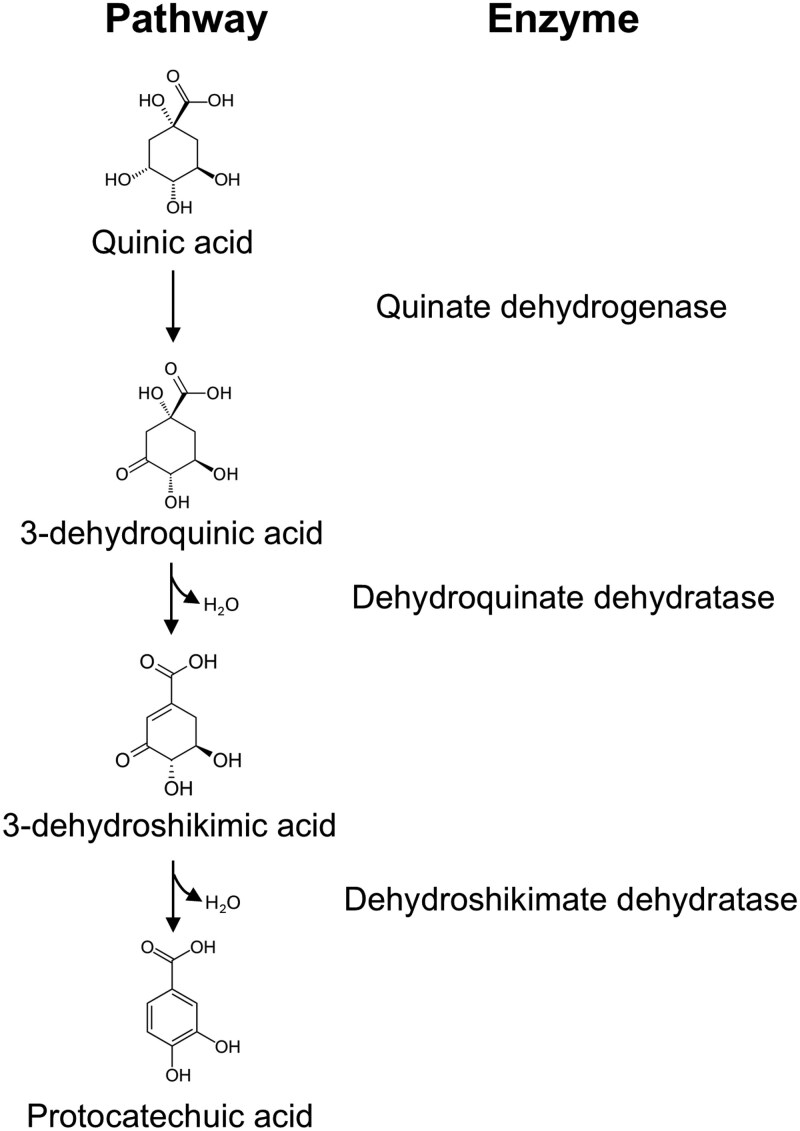
Quinic acid catabolic pathway in fungi.

Many microorganisms are able to utilize quinic acid as a carbon and energy source (Grund and Kutzner 1998; Niu et al. 2017). The initial 3 steps of quinic acid catabolism convert quinic acid to protocatechuic acid, a common intermediate in the degradation of aromatic compounds. The 3 genes involved in this pathway together with 2 regulatory genes were identified in the fungus *Neurospora crassa* and were found to be co-localized. This gene cluster has been foundational for studying transcriptional regulation and the first known gene cluster in eukaryotes containing 2 regulatory genes (Giles et al. 1967; Valone et al. 1971; Giles et al. 1991; Chaleff 2000). The *N. crassa* gene cluster includes 3 structural genes, *qa-3*, *qa-2*, and *qa-4*, respectively, encoding the enzymes quinate dehydrogenase, which converts quinic acid to 3-dehydroquinic acid, dehydroquinate dehydratase, which converts 3-dehydroquinic acid to 3-dehydroshikimic acid, and dehydroshikimate dehydratase, which completes the conversion to protocatechuic acid (Giles et al. 1991). This pathway is shown in Fig. 1. Two regulatory genes named *qa-1f* and *qa-1 s* encode an activator and repressor, respectively, which together control expression of the cluster (Huiet 1984). Sequencing of the gene cluster and DNA-RNA hybridization studies identified 2 additional structural genes, *qa-x* and *qa-y*, which are transcribed upon growth on quinic acid (Patel et al. 1981).

The *qa* gene cluster is conserved in many fungi including *Aspergillus nidulans*, where it is called the *QUT* cluster and contains orthologs of all *qa* genes and one additional gene (*qutH*) of unknown function (Giles et al. 1991; Lamb et al. 1992). The role of *qa-x* and its ortholog in *A. nidulans*, *qutG*, in quinic acid utilization is unknown, as is the function of *qutH*, which has no ortholog in *N. crassa* (Giles et al. 1991; Lamb et al. 1992). In *A. nidulans*, *qutD*, the ortholog of the *qa-y* gene, encodes a protein classified as a member of the “Sugar and other transporters” PFAM family (PF00083) (de Vries et al. 2017). Mutants of *qutD* lose the ability to grow using quinic acid as a carbon source at pH 6.5, suggesting that QutD is a quinate permease. Since mutants of *qutD* grow well in quinic acid media at pH 3.5 but not at pH 6.5, Whittington et al. (1987) suggested that quinate, having an apparent pKa of 3.6, is sufficiently protonated at pH 3.5 to enter the cell by diffusion, while at neutral pH the quinic acid will be deprotonated and requires the QutD permease for transport.

In *Aspergillus niger*, orthologous activator/repressor systems have been shown to regulate the utilization of galacturonic acid and gallic acid (Niu et al. 2017; Arentshorst et al. 2021). Based on the presence of homologous activator/repressor genes, gene organization, and sequence similarity, Niu et al. (2017) predicted that a cluster of genes on Chromosome_8 of *A. niger* is involved in quinate utilization. However, the *A. niger* gene cluster is missing the *qa-3* and *qa-x* orthologs of *N. crassa*, suggesting the genomic organization and regulation of the orthologs of the *N. crassa qa*-clustered genes is potentially different in *A. niger*.

*A. niger* is commonly used industrially to produce enzymes and organic acids as well as in studies of fungal physiology and gene regulation. High-quality genome sequences and annotation (Pel et al. 2007; Andersen et al. 2011; Aguilar-Pontes et al. 2018; Vesth et al. 2018) along with efficient genome-editing tools (Nødvig et al. 2018; Song et al. 2018) are available for *A. niger*. The availability of well-annotated genomes and tools for analyzing global gene expression can provide us with new insights into quinate utilization in *A. niger* that were not possible decades ago during the studies in *N. crassa* and *A. nidulans*. This new knowledge may lead to improved valorization of the common plant metabolite quinic acid that could be used by *A. niger* as an industrial platform to generate valuable compounds.

In this study, we used transcriptomics and mutational analysis to determine the genes involved in the quinic acid utilization of *A. niger*. Our results showed that not all quinate utilization genes are linked. Furthermore, our results led to the discovery in *A. niger* of 2 quinate permease genes; an in-cluster *qupA* is required for optimal growth in quinic acid at neutral pH and an off-cluster *qupB* for optimal growth at acidic pH.

## Materials and methods

### Strains and growth conditions

Table 1 lists the strains used and created in this study. Strain CBS 138852 (N402, *pyrG^−^*, Δ*kusA*) (Kun et al. 2020) was used as the parental strain for all mutants except Δ*qutX*. Strain CBS 138852 was derived from strain N593, which is auxotrophic for uridine (Goosen et al. 1987), and carries a deletion of the *kusA* gene, which has been shown to increase efficiency of homologous recombination in *A. niger* (Meyer et al. 2007). Strain MA234.1 (N402, Δ*kusA*::*amdS*) (Alazi et al. 2016) was used as the parental strain for the isolation of Δ*qutX* mutants. Both strains CBS 138852 and MA234.1 originate from the same parent, N402 (NRRL3→CBS 120.49→N400 cspA1) (Goosen et al. 1987), but they carry different selectable markers, *pyrG* vs *amdS*.

**Table 1.** *A. niger* strains used in this study.

Strain	Genotype	Source
CBS 138852	N402 *pyrG^−^*, Δ*kusA*	Kun et al. (2020)
MA234.1	N402 Δ*kusA*::*amdS*	Alazi et al. (2016)
MA587.1	Δ*qutX* in MA234.1	This study
MS11	Δ*qutR* in CBS 138852	This study
MS12	Δ*qdhA* in CBS 138852	This study
MS13	Δ*dqdA* in CBS 138852	This study
MS14	Δ*dsdA* in CBS 138852	This study
MS15	Δ*qupA* in CBS 138852	This study
MS16	Δ*qupB* in CBS 138852	This study
MS17	Δ*qupA,* Δ*qupB* in CBS 138852	This study

Transformants were grown at 30 °C for 5 d on selective minimal media plates (Hill and Kafer 2001) lacking uridine or uracil (for CBS 138852 and 100 µg/mL hygromycin for MA234.1). For extraction of genomic DNA to be used for PCR screening, 250 μL complete media (minimal media supplemented with 5 g/L yeast extract, 10 mM uridine, and 1 g/L casamino acids) were inoculated with spores and grown for 18 h at 30 °C. Phenotypic testing was performed by spotting 2 µL of fresh spores in a 5 × 10^6^ spore/mL suspension on minimal media plates with 2% (w/v) of either fructose or quinic acid as the sole carbon source and pH adjusted to 3.5 or 6.5 using NaOH or HCl. Testing was performed at least twice using the parental strain and each mutant for each gene. The DH5α strain of *Escherichia coli* was used for maintenance and propagation of plasmids.

Bioreactor fermentation was performed by inoculating 5 L of minimal media containing 0.75% fructose as the sole carbon source and 1.5 mL of 10% (w/v) filter-sterilized yeast extract with 7 × 10^8^ spores/L. Experiments were performed in duplicate using biological replicates of the same strains. Growth occurred at 30 °C and maintained at pH 3 by the addition of titrants (2 M NaOH or 1 M HCl). Sterile air was supplied at 1 L/min. For the first 6 h, stirrer speed was 250 rpm and the culture was aerated using the headspace. Stirrer speed was then increased to 750 rpm, 0.5 mL of the antifoam agent polypropylene glycol P2000 was added, and air was supplied using the sparger. Culture media was harvested at regular intervals and the biomass collected using vacuum filtration with glass microfiber filters (Whatman, Maidstone, UK). The biomass and filtrate were quickly frozen and stored at −80 °C prior to lyophilization. Dry biomass concentrations were determined gravimetrically by comparison to the mass of the culture media. Mycelia that were grown until the mid-exponential phase were used for preparation for RNA.

### RNA sequencing and transcriptomics

RNA-seq data for the control strains obtained from the bioreactor culture samples grown in fructose media were previously deposited in the Sequence Read Archive under accession number SRP078485. RNA-seq data obtained from the bioreactor culture samples grown in quinic acid media as well as the Δ*qutX* mutant cultures were deposited in the Sequence Read Archive under accession number SRP573469. Raw sequence reads were preprocessed using the BBMap package (Bushnell 2014) to trim sequencing adapters and remove reads from ribosomal RNA and phiX. The RSubread software (Liao et al. 2019) was then used to align the preprocessed RNA-seq data to the *A. niger* NRRL3 curated genes. *P*-values were determined using a 2-tailed Welch's *t*-test.

### Construction of mutants using CRISPR/Cas9

Deletion of the repressor *qutX* (*NRRL3_11039*) gene was done using the split-marker technique with the *hph* gene as a selectable marker, as shown in Supplementary Fig. 1 (van den Berg and Maruthachalam 2015). Deletion of *qutX* was confirmed by Southern blot analysis, as shown in Supplementary Fig. 2. CRISPR/Cas9 was used to mutate the other target genes as described (Nødvig et al. 2018; Song et al. 2018). Guide RNA cassettes were inserted into the plasmid ANEp8-Cas9 using ligation-independent cloning (LIC) (Aslanidis and de Jong 1990) for plasmids targeting *NRRL3_11036* (*qupA*) and *NRRL3_05631* (*qupB*). ANEp8-Cas9 plasmids with guide RNA cassettes for *NRRL3_11038* (*qutR*), *NRRL3_08520* (*qdhA*), *NRRL3_11035* (*dqdA*), and *NRRL3_11037* (*dsdA*) were assembled by Integrated DNA Technologies (IDT, Coralville, IA, United States). CRISPR/Cas9 target sequences were identified using Geneious R9.1 (http://www.geneious.com; Kearse et al. 2012). Single-stranded 60-base gene-editing oligonucleotides were co-transformed along with the CRISPR plasmid. These oligonucleotides with 30 bases of homology on each side of the cut site were used to repair the double-stranded breaks and create the desired deletions, as shown in Supplementary Figs. 3 to 7. Supplementary Table 1 lists all primers, oligos, and target sequences used in this study.

### Preparation of protoplasts, gene transformation, and PCR verification

Protoplasts of *A. niger* used for gene transformation was performed using young hyphae according to a previously described method (Debets and Bos 1986). Protoplasts were transformed as previously described (Master et al. 2008) with 1.5 µg of CRISPR plasmid and 1 nmol of gene-editing oligonucleotide per target gene. Deletions were verified by PCR using the primers listed in Supplementary Table 1. Two independent mutants were obtained for each permease knockout including the double knockout, and 3 independent mutants were obtained for the remaining target genes.

### Growth profile of mutants

In addition to examining growth on agar plates, we monitored growth of the permease mutants in liquid media. Minimal media at different pH containing 1% (w/v) quinic acid as the sole carbon source was added to wells of a clear, flat-bottom 96-well plate. Wells were then inoculated with 10 µL of spore solution to a final volume of 200 µL and concentration of 2.5 × 10^5^ spores mL^−1^. Growth occurred for 120 h at 30 °C and was measured by absorbance at 595 nm every hour using a Tecan Sunrise microplate reader (Tecan, Switzerland). Results are the average of triplicates for each strain at each pH level.

## Results

### Quinic acid utilization gene cluster in *A. niger*

**Fig. 2.**
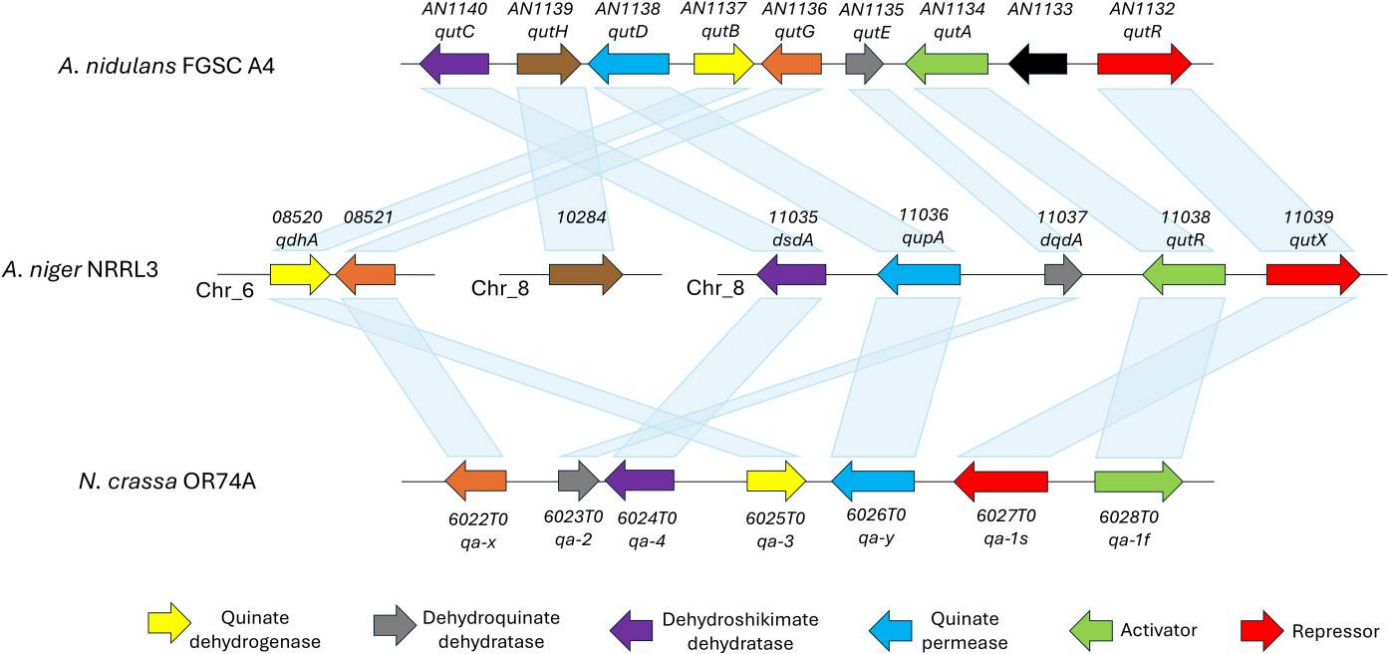
Organization of quinic acid utilization genes in *N. crassa*, *A*. *nidulans*, and *A. niger*. Gene organization data were obtained from the JGI MycoCosm (https://mycocosm.jgi.doe.gov/mycocosm/home; Nordberg et al. 2014). Gene IDs represent NRRL3 ID in *A. niger* (Aguilar-Pontes et al. 2018), AN ID in *A. nidulans* (Broad Institute and Eurofungbase), and NCU ID in *N. crassa* (Broad Institute). Arrows represent direction of transcription.

As discussed by Niu et al. (2017), an apparent quinic acid utilization gene cluster can be found on Chromosome_8 of *A. niger* based on synteny and orthology with *A. nidulans* and *N. crassa*. This cluster has not been studied in detail in *A. niger* and appears to include the activator and repressor, *NRRL3_11038* and *NRRL3_11039*, respectively, seen in *N. crassa* and *A. nidulans* (Valone et al. 1971). We used BLASTP to identify the orthologues in *A. niger* of the characterized quinic acid utilization genes in *N. crassa* and *A. nidulans*. Supplementary Table 2 shows the percentage of sequence identity of the orthologs (>40% identity and >70% for query coverage). Based on the highest sequence identity and partial synteny and gene expression levels (discussed below), we name the following genes as the *A. niger* orthologs: *qutR* (*NRRL3_11038*) encoding the regulator/activator, *qutX* (*NRRL3_11039*) encoding the repressor, *qdhA* (*NRRL3_08520*) encoding quinate dehydrogenase, *dqdA* (*NRRL3_11037*) encoding dehydroquinate dehydratase, *dsdA* (*NRRL3_11035*) encoding dehydroshikimate dehydratase, and *qupA* (*NRRL3_11036*) encoding quinate permease. Table 2 lists *N. crassa* and *A. nidulans* quinic acid cluster genes and their *A. niger* orthologs. Figure 2 shows the gene organization between the *qa* cluster of *N. crassa*, the *QUT* cluster in *A. nidulans*, and their orthologs in *A. niger*.

**Table 2.** Genes of the quinic acid utilization gene cluster in *N. crassa* and their orthologs in *A. nidulans* and *A. niger.*

Gene	*N. crassa* gene	*A. nidulans* ortholog	*A. niger* ortholog
Quinate dehydrogenase	*qa-3*	*qutB*	*qdhA*
Dehydroquinate dehydratase	*qa-2*	*qutE*	*dqdA*
Dehydroshikimate dehydratase	*qa-4*	*qutC*	*dsdA*
Activator	*qa-1f*	*qutA*	*qutR*
Repressor	*qa-1s*	*qutR*	*qutX*
Permease	*qa-y*	*qutD*	*qupA*
Unknown function	*qa-x*	*qutG*	*NRRL3_08521*
Unknown function		*qutH*	*NRRL3_10284*

*A. niger* orthologs were based on sequence identity compared to *N. crassa*, or to *A. nidulans* when no ortholog was found in *N. crassa*.

The *A. niger* orthologs of 5 of the 8 genes in the *QUT* cluster of *A. nidulans* are co-localized on Chromosome_8. This *A. niger* gene cluster includes *dqdA*, *dsdA*, *qupA*, *qutR*, and *qutX*. Note that *qdhA* and *NRRL3_08521* (ortholog of *qutG* of *A. nidulans* and *qa-x* of *N. crassa*) are off-cluster and co-localized on Chromosome_6 of *A. niger*. As well, the ortholog of the *A. nidulans qutH* (*NRRL3_10284*) is also off-cluster. While the function of the *A. nidulans qutG* and *qutH* genes in quinic acid utilization is unknown, the ortholog of the *A. niger qdhA* gene has been shown to encode quinate dehydrogenase (Hawkins et al. 1982). Hence, the synteny analysis reveals that not all the genes involved in quinic acid utilization in *A. niger* are co-localized.

### Transcriptome analysis shows upregulation of quinate utilization genes

Differences in the organization of the quinate cluster in the 3 species potentially provide us with the opportunity to distinguish the clustered genes involved in quinic acid utilization from those that are fortuitously located in the cluster. To identify genes that are upregulated in the presence of quinic acid in *A. niger*, we performed RNA sequencing using cultures grown by batch fermentation in a bioreactor at pH 3.0 with either fructose or quinic acid as the sole carbon source. Further, mutants lacking the repressor-encoding gene *qutX* are expected to constitutively express the quinic acid utilization genes. We constructed a deletion mutant of *qutX* and cultured it on fructose as the sole carbon source for comparison. Table 3 shows the expression levels of the predicted quinic acid cluster genes on quinic acid compared to fructose and their expression in the Δ*qutX* strain compared to the parental strain on fructose. Full transcriptome results for all cultures are shown in Supplementary Table 3.

**Table 3.** RNA-sequencing data for candidate quinic acid utilization genes and their paralogs and for protocatechuic acid utilization genes.

Description	Gene name/ID	Fructose TPM	Quinic acid TPM	Δ*qutX* TPM	Protocatechuic acid TPM
Quinic acid utilization genes and their paralogs					
Quinate dehydrogenase	*qdhA*	10.8	472.5	1611.4	22.1
	*11273*	61.6	21.1	81.4	81.6
Dehydroquinate dehydratase	*dqdA*	1.4	849.5	4196.5	3.1
Dehydroshikimate dehydratase	*dsdA*	5.2	209.1	2186	12.5
	*7603*	1	0.6	1	1.5
Activator	*qutR*	11.1	17.6	84.6	30
Repressor	*qutX*	9.6	24.6	0	28.8
	*8276*	15.5	11.4	29.5	38.9
Permease	*qupA*	7.7	44.1	19.7	52.2
	*qupB*	10	242.1	555	2.34
Unknown function	*8521*	45.8	18.6	62	29.65
	*1575*	54.7	28.1	50	122.2
	*10284*	2.6	16.3	644.8	3.69
Protocatechuic acid utilization genes					
Protocatechuate 3,4-dioxygenase	*prcA*	2.5	2112.4	1079.2	4346.1
Carboxy-*cis,cis*-muconate cyclase	*cmcA*	24.5	219.3	144.8	325.9
3-Carboxymuconolactone hydrolase/decarboxylase	*chdA*	6.9	98.1	40.3	233.1
Unknown function	*837*	10.1	71	21.4	103.6
β-Ketoadipate:succinyl-CoA transferase	*kstA*	41.1	674.2	991.4	709
β-Ketoadipyl-CoA thiolase	*kctA*	16.5	411.8	898.3	499.9

Expression levels were measured as transcripts per million (TPM). *A. niger* strain N402 was used for the cultures grown in media containing fructose, quinic acid, or protocatechuic acid as the sole carbon source. Strain MA587.1 (which has a Δ*qutX* mutation) was used for the Δ*qutX* cultures grown on fructose media. NRRL3 gene IDs are given for unnamed genes. Full RNA-seq data including standard deviations and fold-change values are shown in Supplementary Table 3. Protocatechuic acid pathway genes were identified, and RNA-seq data on protocatechuic acid were obtained from previous research (Sgro et al. 2023).

As expected, most of the genes predicted to be involved in quinic acid utilization were highly upregulated on quinic acid compared to fructose (Table 3). The gene predicted to encode dehydroquinate dehydratase, *dqdA*, was the most highly upregulated on quinic acid with a mean TPM over 600-fold higher compared to fructose and 2970-fold higher in the Δ*qutX* strain on fructose compared to the parent strain on fructose. The genes encoding quinate dehydrogenase, *qdhA*, and dehydroshikimate dehydratase, *dsdA*, were also highly upregulated on quinic acid, 43-fold and 40-fold, respectively, and in the Δ*qutX* strain, 149-fold and 419-fold, respectively. The paralogs of *qdhA* and *dsdA*, *NRRL3_11273* and *NRRL3_07603*, respectively (Table 3), did not display differential upregulation. The activator gene *qutR* was insignificantly upregulated on quinic acid but was upregulated over 7-fold in the Δ*qutX* mutant.

The ortholog of the *A. nidulans qutG* gene (*qa-x* of *N. crassa*) with unknown function, *NRRL3_08521*, was the only ortholog of a member of both the *N. crassa* and *A. nidulans* clusters that displayed no upregulation on both quinic acid and in the Δ*qutX* mutant cultures compared to fructose (Table 3).The paralog of this gene, *NRRL3_01575*, also displayed no upregulation. Mutants with a loss of function of the *qa-x* gene in *N. crassa* have been shown to be able to grow on quinic acid as the sole carbon source (Giles et al. 1991). However, the ortholog of the *A. nidulans qutH*, the second gene in the cluster with unknown function, *NRRL3_10284*, was upregulated on quinic acid and in the Δ*qutX* mutant, 6-fold and 245-fold, respectively.

In addition to the quinate cluster, all genes known to be involved in the catabolism of protocatechuic acid in *A. niger* (Sgro et al. 2023), the intermediate metabolite of quinic acid degradation, were also found to be highly expressed and upregulated in all samples grown on quinic acid and in the Δ*qutX* strain. Transcriptome results for the protocatechuic acid catabolism genes are shown in Table 3.

### Off-cluster paralog of quinate permease

In addition to the quinate permease encoded by the in-cluster gene *qupA*, a BLASTP search using the sequence of the *N. crassa* permease qa-y revealed a potential second quinate permease encoded by *NRRL3_05631*, which we name *QupB*. The *qupB* gene is located on Chromosome_4 and not linked to the other genes involved in quinic acid utilization. QupB has 66% sequence identity to QupA and a similar length (536 amino acids compared to 539 for QupA).

Transcriptome data provided additional evidence that *qupB* encodes a second quinate permease, as it was even more highly expressed on quinic acid than *qupA.* Although both were upregulated, *qupB* was expressed and upregulated over four times as much as *qupA* (24-fold vs 5.7-fold) on quinic acid. In the Δ*qutX* strain, *qupB* was 56-fold upregulated while *qupA* was only 2.5-fold upregulated (Table 3).

### Knockout mutants of quinic acid pathway genes display growth defects

**Fig. 3.**
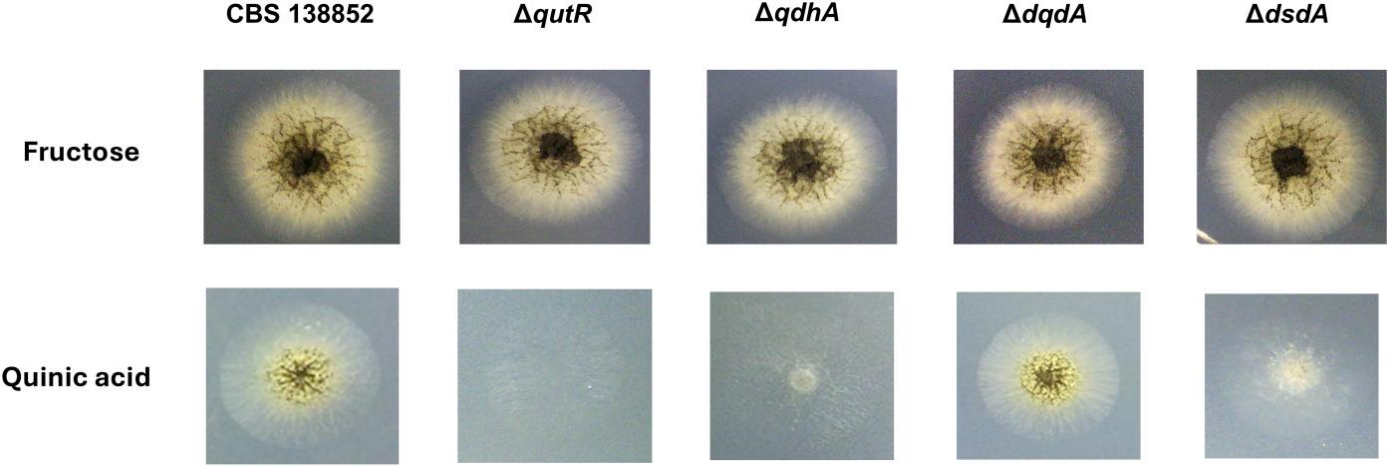
Growth phenotype of parent and quinic acid pathway activator and enzyme mutants. Parental (CBS 138852) and mutant strains were grown for 3 d at 30 °C on minimal media with 2% fructose or 2% quinic acid as sole carbon source.

Mutants in *A. niger* were created using CRISPR-Cas9 to individually delete *qutR*, *qdhA*, *dqdA*, and *dsdA*. Successful deletions in these genes were verified using PCR (Supplementary Figs. 3 to 6 and Table 1). These mutants were grown on minimal media agar plates using quinic acid as the sole carbon source to observe the growth phenotype (Fig. 3). The phenotypes of 2 additional independently isolated mutants for each gene are shown in Supplementary Fig. 8. Mutants of all four genes grew equally well compared to the parental strain CBS 138852 on fructose media, but growth was impaired on quinic acid media to varying degrees. The Δ*qutR* and Δ*qdhA* mutants grew extremely poorly and failed to sporulate on quinic acid. The Δ*dsdA* mutant growth was also poor and had minimal sporulation. These results are similar to those observed in *N. crassa* and *A. nidulans*. However, unlike in those species, the Δ*dqdA* mutant in *A. niger* appeared to grow as well as the parental strain. Table 4 shows a summary of the phenotypes of the quinate pathway mutants of *A. niger, N. crassa* and *A. nidulans*. Mutants of the genes encoding the enzymes involved in the catabolism of protocatechuic acid, constructed in a previous study (Sgro et al. 2023), were also confirmed to display impaired growth on quinic acid media (Supplementary Fig. 9).

**Table 4.** Comparison of the growth phenotypes observed in knockout mutants of genes involved in quinic acid utilization in *N. crassa*, *A. nidulans*, and *A. niger*.

Gene	*N. crassa* gene	*A. nidulans* ortholog	*A. niger* ortholog
Quinate dehydrogenase	*qa-3* No growth (Case et al. 1978)	*qutB* No growth (Hawkins et al. 1982)	*qdhA* Very weak growth
Dehydroquinate dehydratase	*qa-2* No growth (Chaleff 2000)	*qutE* No growth^a^ (Lamb et al. 1991)	*dqdA* Normal growth
Dehydroshikimate dehydratase	*qa-4* No growth (Chaleff 2000)	*qutC* Weak growth (Hawkins et al. 1982)	*dsdA* Weak growth
Activator	*qa-1f* No growth (Valone et al. 1971)	*qutA* No growth (Hawkins et al. 1982)	*qutR* Very weak growth
Repressor	*qa-1s* Normal growth (Case et al. 1992)	*qutR* Normal growth (Grant et al. 1988)	*qutX* Normal growth
Permease	*qa-y* No growth (Geever et al. 1989; Case et al. 1992)	*qutD* No growth at pH 6.5 Normal growth at pH 3.5 (Whittington et al. 1987)	*qupA/qupB* pH-dependent growth^b^ Very weak in double mutant

^a^Growth was observed in mutants with a 5-fold overexpression of the pentafunctional AROM protein.

^b^Mutants lacking *qupA* had weak growth at pH 6.5 and normal growth at pH 3.5. Mutants lacking *qupB* had normal growth at pH 6.5 and weak growth at pH 3.5.

### Mutational analysis reveals uptake of quinate is facilitated by 2 permeases

**Fig. 4.**
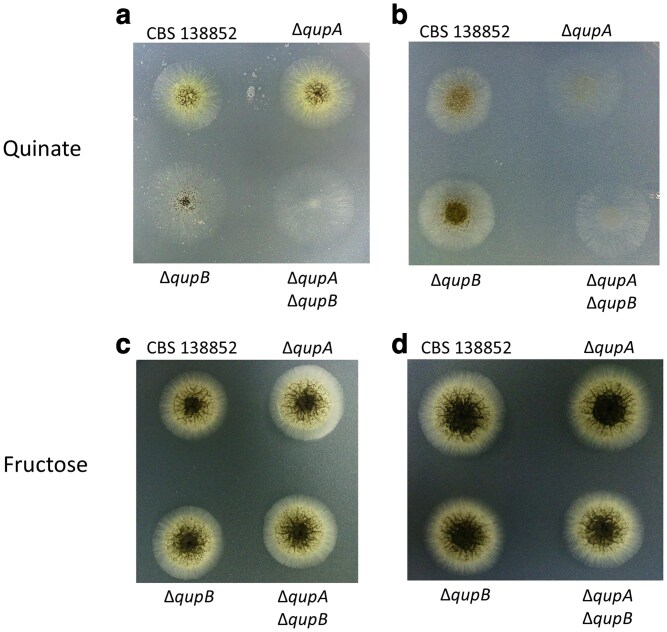
Growth phenotype of parent strain (CBS 138852) and permease mutants. Spores were spotted on plates and grown at 30 °C for 3 d on minimal media with 0.5% quinate as the sole carbon source at pH 3.5 a) and pH 6.5 b), or with 0.5% fructose at pH 3.5 c) and pH 6.5 d).

Three *A. niger* quinate permease mutants were created using CRISPR-Cas9 to inactivate *qupA* and *qupB* both individually and together. Successful deletions in these genes were verified using PCR (Supplementary Fig. 7; Supplementary Table 1). In *A. nidulans*, mutants of the quinate permease, QutD, have been reported to grow at pH 3.5 but not at pH 6.5 (Whittington et al. 1987). We therefore tested the growth phenotypes of the Δ*qupA,* Δ*qupB*, and Δ*qupA*/Δ*qupB* double mutants on minimal media agar plates with quinic acid as the sole carbon source at pH 3.5 and 6.5 (shown in Fig. 4a and b, respectively). Results for the *qupA* mutants were consistent with this gene being an ortholog of the *qutD* gene of *A. nidulans.* Growth at pH 3.5 was equal to that of the parent strain, while growth at pH 6.5 was significantly reduced. Conversely, the *qupB* mutants exhibited poor growth at pH 3.5 but strong growth equal to the parent strain at pH 6.5. A double knockout strain lacking expression of both permeases grew very poorly at both pH levels and was unable to sporulate after 4 d at 30 °C. Table 4 shows a comparison of these results to the phenotypes of permease mutants in *N. crassa* and *A. nidulans.* The phenotypes of one additional independently isolated mutant for each permease gene and the double knockout mutant are shown in Supplementary Fig. 10.

All strains were also grown on minimal media plates with fructose as the sole carbon source at both pH levels as a control for unexpected general effects on growth at different pH levels. Quinate permeases have no known or expected effect on fructose uptake or metabolism, and transcription of these genes is expected to be very low when grown on carbon sources other than quinic acid (Table 3). In the absence of quinic acid, the repressor protein is thought to bind to the activator protein causing the quinic acid utilization genes to be expressed at very low levels (Giles et al. 1991). In addition, the *N. crassa* quinate permease has been shown to have a role in catabolite repression, with transcription being more strongly repressed in the presence of preferred carbon sources than other *qa* genes (Arnett et al. 2009). As expected, all mutant strains grew as strongly as the parent on fructose media at both pH 3.5 and 6.5 (Fig. 4c and d, respectively).

**Fig. 5.**
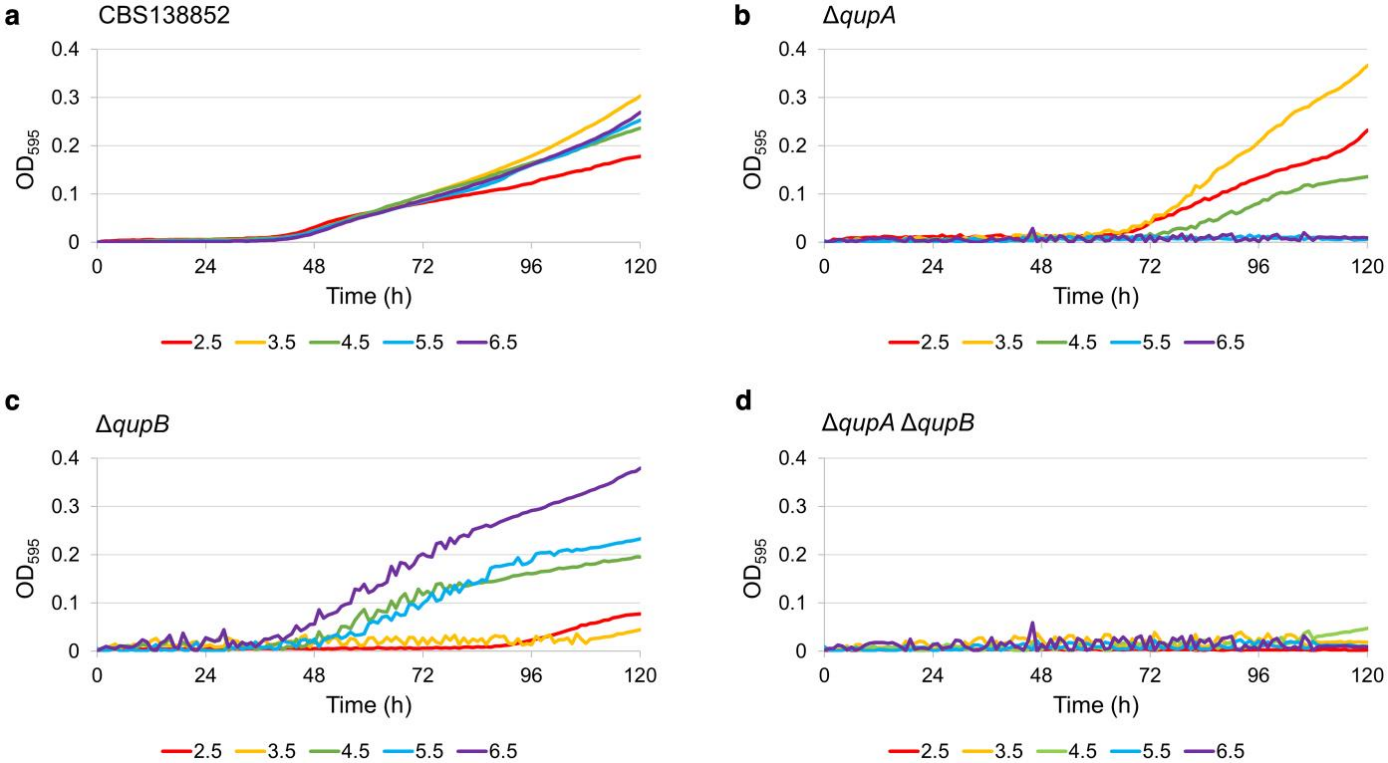
Growth profile of parental strain (CBS 138852) and permease mutants in liquid media with quinate as sole carbon source at different pH levels. Samples are the average of 3 replicates. Measurements were taken every hour.

To determine the activity of the 2 permeases over a range of pH values, growth assays were performed by measuring absorbance at 595 nm in a microplate reader. The parent strain and each of the 3 mutant strains were grown in liquid media with quinic aid as the sole carbon source for 5 d and measurements were taken every hour. There was minimal effect on growth in the parent strain caused by differences in pH 3.5 to 6.5 with reduced growth at pH 2.5 (Fig. 5a). However, the mutant strain lacking expression of *qupA* (Fig. 5b) grew optimally at pH 3.5, with weaker growth at pH 2.5 and 4.5, and no growth at pH 5.5 or 6.5. The *qupB* mutant strain (Fig. 5c) had an overlap in function only at pH 4.5. This mutant grew optimally at pH 6.5, with weaker growth at pH 5.5 and 4.5, and little to no growth at pH 3.5 or 2.5. The double mutant strain (Fig. 5d) did not grow at any pH level. These results, along with Fig. 4, suggest that there is not a significant amount of quinic acid entering the mycelium through either diffusion or any other transporters.

## Discussion

The quinic acid utilization gene cluster and catabolic pathway have been previously identified and characterized in the filamentous fungi *N. crassa*. Using orthology, transcriptomics, and mutational analysis, we have identified the genes in *A. niger* encoding the 3 enzymes of the quinic acid catabolic pathway: *qdhA*, *dqdA*, and *dsdA*, encoding quinate dehydrogenase, dehydroquinate dehydratase, and dehydroshikimate dehydratase, respectively. We have also identified the genes encoding the activator and repressor, *qutR* and *qutX*, respectively, and the quinate permease, *qupA*. Our results have provided new insights into quinic acid utilization in fungi. Unlike the quinic acid gene clusters in *N. crassa* and *A. nidulans*, the *qdhA* gene of *A. niger* is not linked to the activator-repressor and catabolic genes. Therefore, the co-localization of the activator-repressor genes and the catabolic genes under their control is not a requirement for gene regulation, but a result of genome evolution. The absence of upregulation on quinic acid of *NRRL3_08521*, an ortholog of *qa-x* of *N. crassa* and *qutG* of *A. nidulans*, lends further support that this gene is not involved in quinic acid utilization. Notably, we identified a second quinate permease which functions at a different pH than the previously characterized permease.

*A. niger* contains 3 similar activator-repressor modules to *qutR*/*qutX* of the quinic acid pathway: *gaaR*/*gaaX* (*NRRL3_08195*/*NRRL3_08194*), which regulates the genes involved in the utilization of galacturonic acid (Niu et al. 2017); *tanR*/*tanX* (*NRRL3_08275*/*NRRL3_08276*), which regulates the genes involved in tannic acid degradation and gallic acid utilization (Arentshorst et al. 2021); and *NRRL3_07604*/*NRRL3_07605*, which regulate genes with currently unknown function. Although the genes encoding the activator and repressor are linked for all these modules, the enzyme-encoding genes for the galacturonic acid and tannic acid catabolic pathways are not linked to each other or to their respective activator-repressor genes (Niu et al. 2017; Arentshorst et al. 2021). This provides further evidence that linkage between the regulatory and catabolic genes is not required for gene regulation.

In the activator-repressor module, the repressor is predicted to interact with the activator under noninducing conditions and inhibits the expression of target genes, while in the presence of quinic acid or other pathway intermediates, binding of one or more of these compounds to the repressor causes an allosteric change which negates its function on the activator (Lamb et al. 1996; Arentshorst et al. 2021). As a result, loss of function of the activator should cause a severely reduced ability to grow on quinic acid while loss of function of the repressor should cause constitutive expression of the target genes of the activator. These expected results have been seen in *N. crassa* (Geever et al. 1989) and *A. nidulans* (Levett et al. 2000) (Table 4). Here, we confirm the role of these genes by showing that Δ*qutR* displays severe growth defects on quinic acid (Fig. 3) and that Δ*qutX* results in the upregulation of quinate catabolic genes on fructose, a noninducing carbon source (Table 3). Both *qutR* and *qutX* appear to be upregulated by quinic acid, though insignificantly and to a lesser degree than the structural genes. The low level of upregulation can be explained by the interaction between QutR and QutX, which occurs at the posttranslational level and not the transcriptional level (Lamb et al. 1996; Arentshorst et al. 2021).

The Δ*qdhA* mutant showed severe growth impairment on quinic acid media and the Δ*dsdA* mutant showed reduced growth and sporulation compared to the parent strain. These results are similar to what was observed in previous experiments in *N. crassa* and *A. nidulans* (Table 4). The remaining growth in the Δ*dsdA* strain may be supported by the activity of another gene with a similar function. The potential candidate would be its paralog *NRRL3_07603* (Supplementary Table 2). However, *NRRL3_07603* is minimally expressed under any of the growth conditions of this study (Table 3), making it unlikely to be involved in the reduced growth of Δ*dsdA* on quinic acid. The *dqdA* deletion did not appear to have an effect on growth on quinic acid in *A. niger*, while this deletion prevented growth in *N. crassa* and *A. nidulans* (Table 4). Although *dqdA* was the only dehydroquinate dehydratase identified in *A. niger* based on homology to *N. crassa* or *A. nidulans*, a different dehydroquinate dehydratase is one of the 5 functional domains of the pentafunctional AROM protein, which catalyzes 5 enzymatic steps of the shikimate pathway involved in aromatic amino acid biosynthesis (Duncan et al. 1987). This dehydroquinate dehydratase domain of the *A. niger* AROM protein (NRRL3_11273) shows no significant similarity to DqdA, but as they catalyze the same enzymatic reaction, it may explain why the *dqdA* knockout mutation had no effect on the growth phenotype on quinic acid (Fig. 3). This was observed with the DqdA ortholog in *A. nidulans*, QutE, although the QutE mutant was only able to grow when the AROM protein was overexpressed 5-fold (Lamb et al. 1991).

The *A. niger* ortholog of the *qa-x* gene in *N. crassa* and *qutG* gene in *A. nidulans*, *NRRL3_08521*, which has an unknown function in those species, was not upregulated by growth on quinic acid. While all known genes involved in this pathway in *N. crassa* and *A. nidulans* are clustered, in *A. niger*, the genes are spread across 3 locations (Fig. 2). The lack of upregulation of the *NRRL3_08521* gene in *A. niger* where it is now located off-cluster likely indicates that the gene is not involved in the quinic acid utilization pathway. This would also explain the lack of growth defect on quinic acid in the loss-of-function *qa-x* mutant in *N. crassa* (Giles et al. 1991). The *A. niger* ortholog of the A*. nidulans qutH* gene, *NRRL3_10284*, has no ortholog in *N. crassa* and is also located off-cluster. However, this gene was upregulated on quinic acid and in the Δ*qutX* mutant. The protein encoded by this gene contains domains in the Gfo/Idh/MocA oxidoreductases family. It is possible this enzyme performs a redox reaction that modulates the activity of one or more of the QdhA, DqdA, or DsdA activities in *A. niger* and *A. nidulans* but is not essential for quinic acid catabolism as indicated by the absence of its encoding gene in *N. crassa*.

The genes of the protocatechuate pathway were identified and characterized in *A. niger* (Sgro et al. 2023). These genes are upregulated on quinic acid, which is expected as protocatechuate is the product of the quinic acid pathway. However, these genes were also upregulated in the Δ*qutX* strain grown on fructose (Table 3). This is likely due to the activation of the transcription regulator of the protocatechuate pathway genes, which has not been identified, by *qutR*/*qutX.* It may also be possible that the promoter regions of the protocatechuate pathway genes contain sequences which bind both *qutR* and the protocatechuate pathway transcription regulator. The protocatechuate pathway cannot be regulated solely by *qutR/qutX* because the quinic acid utilization genes were not highly upregulated when *A. niger* was grown on protocatechuate (Table 3). The slight upregulation observed in some of the quinic acid genes when grown in protocatechuate may be due to a limited ability for protocatechuate to interact directly or indirectly with QutR or the cross-activation of some of these genes by the unknown protocatechuate pathway regulator.

Our results show the presence of a second permease, previously unknown in any species, which functions at a different pH level than the in-cluster permease. *Aspergilli* are known to contain many transporter genes, typically over 1000, with *A. niger* having 1287 genes annotated as transporters. Research by de Vries et al. (2017) compared the genomes of 19 species of *Aspergilli* and 16 of other fungi to estimate the total number of genes in each species which contained transporter-related Pfam motifs. The results showed that even the *Aspergillus* species with the lowest number of transporters (*A. clavatus* with 810) had more than double the amount found in yeasts like *Saccharomyces cerevisiae* and *Schizosaccharomyces pombe* (de Vries et al. 2017). The Major Facilitator Superfamily, which includes the quinic acid permeases, was the group with the largest expansion in the *Aspergilli* compared to yeasts and correlates with the diversity of substrates the species can use as sources of carbon (de Vries et al. 2017). Transporters are known to exist in *A. niger* and other fungi which function on the same substrates but with different affinities or have different substrate specificity (Torres et al. 1996; Sloothaak et al. 2016). Several transporters in *A. niger* and other fungi are also known to be regulated at the transcriptional level in response to pH by the *pacC* gene (Vankuyk et al. 2004). However, transporters in fungi which function on the same substrate at different pH levels are previously unreported. Given the comparatively large number of transporters and the wider pH range tolerated by *Aspergilli* compared to yeasts, it is possible that in addition to an expanded diversity of substrates, there may be other transporters similarly functioning on the same substrate at different pH levels.

The newly discovered permease gene *qupB* has an ortholog in *A. nidulans* (*AN6805.2* in strain FGSC A4), a gene encoding a 549 amino acid protein with 78% identity over the length of the QupB protein. The *A. nidulans* gene is also located outside of its quinic acid cluster. This second permease is likely responsible for the growth at pH 3.5 in *A. nidulans* as it is in *A. niger*. By orthologous analysis, we can identify only one quinate permease in *N. crassa*, perhaps reflecting a narrower range of pH for growth for this species (Cupertino et al. 2012).

## Supplementary Material

jkaf199_Supplementary_Data

## Data Availability

RNA-seq data were deposited in the Sequence Read Archive under accession numbers SRP078485 (Niu et al. 2016) (control strains grown on fructose media) and SRP573469 (Sgro et al. 2025) (strains grown on quinic acid media and the Δ*qutX* mutant strains grown on fructose). Supplemental material available at G3 online.
