# Supplementary material for: An examination of the quinic acid utilization genes in Aspergillus niger reveals the involvement of 2 pH-dependent permeases
Source: G3 (Bethesda). 2025 Aug 25;15(11):jkaf199. doi: 10.1093/g3journal/jkaf199 (PMC12610400; doi:10.1093/g3journal/jkaf199)
Supplement: jkaf199_Supplementary_Data [file jkaf199_supplementary_data.zip › Supplementary_Figure_legends_G3-2025-406129.docx]

**Supplementary Figure legends**

**Supplementary Figure 1** Diagram of split marker method used for replacement of the *qutX* gene with the *hph* gene of *Escherichia coli*. Two oligonucleotides were generated by fusion PCR then transformed together to replace the *qutX* gene with the selectable marker *hph* flanked by the *Aspergillus nidulans* promoter *gpdA* and terminator *trpC* by homologous recombination.

**Alt text:** A diagram of the split marker method showing the genes, PCR products, and primers involved in the replacement of *qutX* with *hph*.

**Supplementary Figure 2** Southern blot analysis to verify replacement of the *qutX* gene with the *hph* gene of *Escherichia coli.* DNA fragments hybridized with two different probes targeting the 5’ and 3’ regions of the *qutX* gene following digestion of genomic DNA by BamHI. Fragment sizes were 2273 bp in the wild type locus (MA234.1 strain) and 2998 bp in the Δ*qutX* strains using the 5’ *qutX* probe. Fragment sizes were 4090 bp in the wild type locus and 3135 bp in the Δ*qutX* strains using the 3’ *qutX* probe. Four independent Δ*qutX* isolates are shown.

**Alt text:** Diagrams of the fragment sizes expected and gel images verifying the replacement of *qutX* with *hph.*

**Supplementary Figure 3** Diagram of gene knockout design and verification of Δ*qutR* mutant. (A) Blue lines show the two homology arms of the rescue template oligonucleotide used to repair the gene and create a 468 base pair deletion. Green arrows indicate primers used for verification of the deletion. (B) Using the verification primers results in a 1374 bp band in the control strain (CBS 138852) and a 906 bp band upon successful deletion in the mutant strains. L = ladder (GeneRuler 1 kb Plus DNA Ladder; Thermo Scientific).

**Alt text:** Diagram showing the *qutR* gene with the primers and expected fragment sizes to verify the deletion, and a gel image showing the expected sizes.

**Supplementary Figure 4** Diagram of gene knockout design and verification of Δ*qdhA* mutant. (A) Blue lines show the two homology arms of the rescue template oligonucleotide used to repair the gene and create a 764 base pair deletion . Green arrows indicate primers used for verification of the deletion. (B) Using the verification primers results in a 1255 bp band in the control strain (CBS 138852) and a 491 bp band upon successful deletion in the mutant strains. L = ladder (GeneRuler 1 kb Plus DNA Ladder; Thermo Scientific).

**Alt text:** Diagram showing the *qdhA* gene with the primers and expected fragment sizes to verify the deletion, and a gel image showing the expected sizes.

**Supplementary Figure 5** Diagram of gene knockout design and verification of Δ*dqdA* mutant. (A) Blue lines show the two homology arms of the rescue template oligonucleotide used to repair the gene and create a 403 base pair deletion . Green arrows indicate primers used for verification of the deletion. (B) Using the verification primers results in a 892 bp band in the control strain (CBS 138852) and a 489 bp band upon successful deletion in the mutant strains. L = ladder (GeneRuler 1 kb Plus DNA Ladder; Thermo Scientific).

**Alt text:** Diagram showing the *dqdA* gene with the primers and expected fragment sizes to verify the deletion, and a gel image showing the expected sizes.

**Supplementary Figure 6** Diagram of gene knockout design and verification of Δ*dsdA* mutant. (A) Blue lines show the two homology arms of the rescue template oligonucleotide used to repair the gene and create a 588 base pair deletion . Green arrows indicate primers used for verification of the deletion. (B) Using the verification primers results in a 1389 bp band in the control strain (CBS 138852) and a 801 bp band upon successful deletion in the mutant strains. L = ladder (GeneRuler 1 kb Plus DNA Ladder; Thermo Scientific).

**Alt text:** Diagram showing the *dsdA* gene with the primers and expected fragment sizes to verify the deletion, and a gel image showing the expected sizes.

**Supplementary Figure 7** Diagram of gene knockout design and verification of permease mutants. (A) Red line indicates the CRISPR-Cas9 cut site. Blue line shows the rescue template oligonucleotide used to repair the gene and cause a 10 base pair deletion . Green arrows indicate primers used for verification of the deletion. (B) Deletion of 10 base pairs from *qupB* was performed the same way but with primers creating a 70 base pair fragment (60 base pairs with deletion). (C) PCR verification of deletions in *qupA* and *qupB*. Forward and reverse primers on either side of both deletion sites were included in all samples. Resulting *qupA* fragments are 100 bp in control (CBS 138852) and 90 bp upon successful deletion. *qupB* fragments are 70 bp in control and 60 bp upon successful deletion. L = ladder (GeneRuler 1 kb Plus DNA Ladder; Thermo Scientific).

**Alt text:** Diagram showing the *qupA* and *qupB* genes with the primers and expected fragment sizes to verify each deletion, and a gel image showing the expected sizes for each single and double deletion.

**Supplementary Figure 8** Growth phenotype of parent and additional independently isolated mutants of the quinic acid pathway activator and enzymes. Parental (CBS 138852) and mutant strains were grown for 3 days at 30°C on minimal media with 2% fructose or 2% quinic acid as sole carbon source.

**Alt text:** Photographs of the growth phenotype of quinic acid pathway mutants showing growth on fructose media and quinic acid media.

**Supplementary Figure 9** Growth Phenotype of protocatechuic acid catabolic pathway mutants on fructose, protocatechuic, and quinic acid media. Strain CBS 138852 is the parent strain used to construct the deletion mutants. The following genes were previously demonstrated to be required for the utilization of protocatechuic acid (Sgro *et al*. 2023): *NRRL3_01405* (*prcA*), *NRRL3_02586* (*cmcA*), *NRRL3_01409* (*chdA*), *NRRL3_00837* (unknown function), *NRRL3_01886* (*kstA*), and *NRRL3_01526* (*kctA*). The gene *NRRL3_08340* is a paralogue of *NRRL3_1409* that is highly upregulated but is not required for growth on protocatechuic acid (Sgro *et al*. 2023). This gene is also highly upregulated on quinic acid (Supplementary Table 3), and as shown not required for growth on quinic acid.

**Alt text:** Photographs of the growth phenotypes of protocatechuic acid pathway mutants on fructose, protocatechuic acid, and quinic acid media.

**Supplementary Figure 10** Growth phenotype of parent strain (CBS 138852) and additional independently isolated permease mutants. Spores were spotted on plates and grown at 30°C for three days on minimal media with 0.5% quinate as the sole carbon source at pH 3.5 (A) and pH 6.5 (B), or with 0.5% fructose at pH 3.5 (C) and pH 6.5 (D).

**Alt text:** Photographs of the growth phenotype of the parent stain and permease mutants on fructose and quinic acid at both pH 3.5 and 6.5.
