# Supplementary material for: An examination of the quinic acid utilization genes in Aspergillus niger reveals the involvement of 2 pH-dependent permeases
Source: G3 (Bethesda). 2025 Aug 25;15(11):jkaf199. doi: 10.1093/g3journal/jkaf199 (PMC12610400; doi:10.1093/g3journal/jkaf199)
Supplement: jkaf199_Supplementary_Data [file jkaf199_supplementary_data.zip › Supplementary_Table_1_G3-2025-406129.docx]

**Supplementary Table 1** Primers, oligos, and CRISPR target sequences used to create and verify deletions in the *A. niger* quinic acid genes.

| **Name** | **Sequence** | **Description** |
| --- | --- | --- |
| tRNA LIC Fw | **CAACCTCCAATCCAATTTG**ACTCCGCCGAACGTACTG | Forward primer binding to tRNA promoter. Sequence used to mediate ligation-independent cloning is shown in bold |
| tRNA LIC Rv | **ACTACTCTACCACTATTTG**AAAAGCAAAAAAGGAAGGTACAAAAAAGC | Reverse primer binding to tRNA terminator. Sequence used to mediate ligation-independent cloning is shown in bold |
| qutXP1f | AAGTCTTGTGTCGCATGGCA | Forward primer for amplification of 5' *qutX* flank |
| qutXP2r | CAATTCCAGCAGCGGCTGTCGTGCACAGGGAATTTTTGT | Reverse primer for amplification of 5' *qutX* flank |
| qutXP3f | ACACGGCACAATTATCCATCGAATGCTCATTGGCCAAGAATATG | Forward primer for amplification of 3' *qutX* flank |
| qutXP4r | AAAGCATGCCTGAATCTCGAA | Reverse primer for amplification of 3' *qutX* flank |
| hygP6f | AAGCCGCTGCTGGAATTGGGCTCTGAGGTGCAGTGGAT | Forward primer for amplification of *Pgpda*-5’ *hph* |
| hygP9r | GGCGTCGGTTTCCACTATC | Reverse primer for amplification of *Pgpda*-5’ *hph* |
| hygP8f | AAAGTTCGACAGCGTCTCC | Forward primer for amplification of 3’ *hph*-*TtrpC* |
| hygP7r | CGATGGATAATTGTGCCGTGTTGGGTGTTACGGAGCATTCA | Reverse primer for amplification of 3’ *hph*-*TtrpC* |
| *qupA* LIC Fw | **ACAAGAGGTGAAGGAGGCAA**GTTTTAGAGCTAGAAATAGCAAG | Forward primer binding to gRNA scaffold. gRNA sequence in bold |
| *qupA* LIC Rv | **TTGCCTCCTTCACCTCTTGT**GACGAGCTTACTCGTTTCG | Reverse primer binding to tRNA promoter. gRNA sequence in bold |
| *qupB* LIC Fw | **GAGTCGGACGATCCTCAACG**GTTTTAGA GCTAGAAATAGCAAG | Forward primer binding to gRNA scaffold. gRNA sequence in bold |
| *qupB* LIC Rv | **CGTTGAGGATCGTCCGACTC**GACGAGCTTACTCGTTTCG | Reverse primer binding to tRNA promoter. gRNA sequence in bold |
| *qupA* RT | **CTACAACTGGCGTATCTACCTCCTGGCGGC**TTCACCTCTTGTATGATCGGCTACGACAGT | Single-stranded oligonucleotide rescue template for mutating *qupA* with 10bp deleted between bold and unbold homology arms |
| *qupB* RT | **TTCTCCCATACAATGGGTGGAATTCTTCAC**ATCGTCCGACTCCCAAAAATGTCTACAATT | Single-stranded oligonucleotide rescue template for mutating *qupB* with 10bp deleted between bold and unbold homology arms |
| *qutR* RT | **CGATGGCGTGCAGCCCGTGTGCTCGACTTG**CGGGGTGCGGATTACGCGAGACTCGGCGAC | Single-stranded oligonucleotide rescue template for mutating *qutR* with 468 bp deleted between bold and unbold homology arms |
| *qdhA* RT | **AATCGCTGCCAATCTACCAACCCGCCCAAC**GACGGCGTTGTTGGGCGCCAAGGACGATGG | Single-stranded oligonucleotide rescue template for mutating *qdhA* with 764 bp deleted between bold and unbold homology arms |
| *dqdA* RT | **CTCCATCTCCCAAAAGAACTCAAACACCAC**GGCGTGCAGGGGTATAGGGTTGCGGTTGAG | Single-stranded oligonucleotide rescue template for mutating *dqdA* with 403 bp deleted between bold and unbold homology arms |
| *dsdA* RT | **GCCCAACCGTCTCGGCATCGCCTCCATGTC**CGCGGAGGCGGATCTGGCTGCGTCGTTGAA | Single-stranded oligonucleotide rescue template for mutating *dsdA* with 588 bp deleted between bold and unbold homology arms |
| *qupA* target sequence | ACAAGAGGTGAAGGAGGCAA**CGG** | CRISPR target sequence with PAM site included in bold |
| *qupB* target sequence | GAGTCGGACGATCCTCAACG**AGG** | CRISPR target sequence with PAM site included in bold |
| *qutR* target sequence | GCTCTTAGCCGTCAATGCCG**CGG** | CRISPR target sequence with PAM site included in bold |
| *qdhA* target sequence | GCACCTGGACGACCTTACCG**AGG** | CRISPR target sequence with PAM site included in bold |
| *dqdA* target sequence | CAACGCATCCCGGATCGCGA**CGG** | CRISPR target sequence with PAM site included in bold |
| *dsdA* target sequence | ATAGATACTCGGTTTGCTTG**CGG** | CRISPR target sequence with PAM site included in bold |
| *qupB* Fw | TTCTCCCATACAATGGGT | Forward primer binding upstream of deletion |
| *qupB* Rv | AATTGTAGACATTTTTGGGA | Reverse primer binding downstream of deletion |
| *qupA* Fw | CTACAACTGGCGTATCTACC | Forward primer binding upstream of deletion |
| *qupB* Rv | GTCGAGGGAGATAGTCGT | Reverse primer binding downstream of deletion |
| *qutR* Fw | GGTAAGAAGTTAGGGCCGG | Forward primer binding upstream of deletion |
| *qutR* Rv | GCGTCGTCTTCATTATGACG | Reverse primer binding downstream of deletion |
| *qdhA* Fw | CCGTACCAACCTGTAGGAG | Forward primer binding upstream of deletion |
| *qdhA* Rv | CCCACAATAACCTTCCATCC | Reverse primer binding downstream of deletion |
| *dsdA* Fw | CCTGTGTGCCGTTACACAG | Forward primer binding upstream of deletion |
| *dsdA* Rv | CCCAGTACTAGCAGCATAGC | Reverse primer binding downstream of deletion |
| *dqdA* Fw | CTCAGTGAGTAAGAGGGAGG | Forward primer binding upstream of deletion |
| *dqdA* Rv | GTGAACTACCCAGAGGAAGG | Reverse primer binding downstream of deletion |
