# Supplementary figures and images for: An examination of the quinic acid utilization genes in Aspergillus niger reveals the involvement of 2 pH-dependent permeases
Source: G3 (Bethesda). 2025 Aug 25;15(11):jkaf199. doi: 10.1093/g3journal/jkaf199 (PMC12610400; doi:10.1093/g3journal/jkaf199)

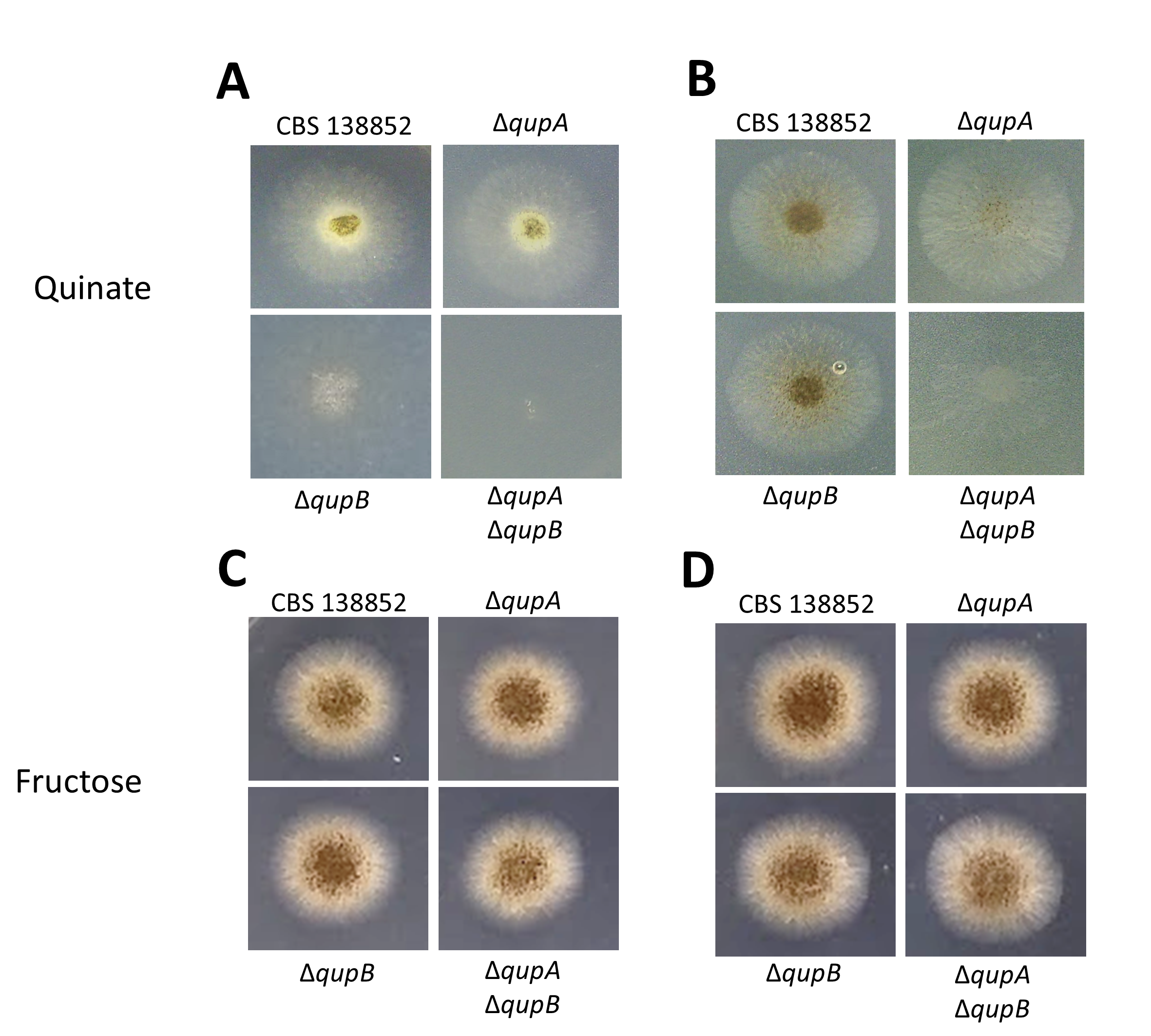

Supplement: jkaf199_Supplementary_Data [file jkaf199_supplementary_data.zip › Supplementary_Figure_10_G3-2025-406129.tif]

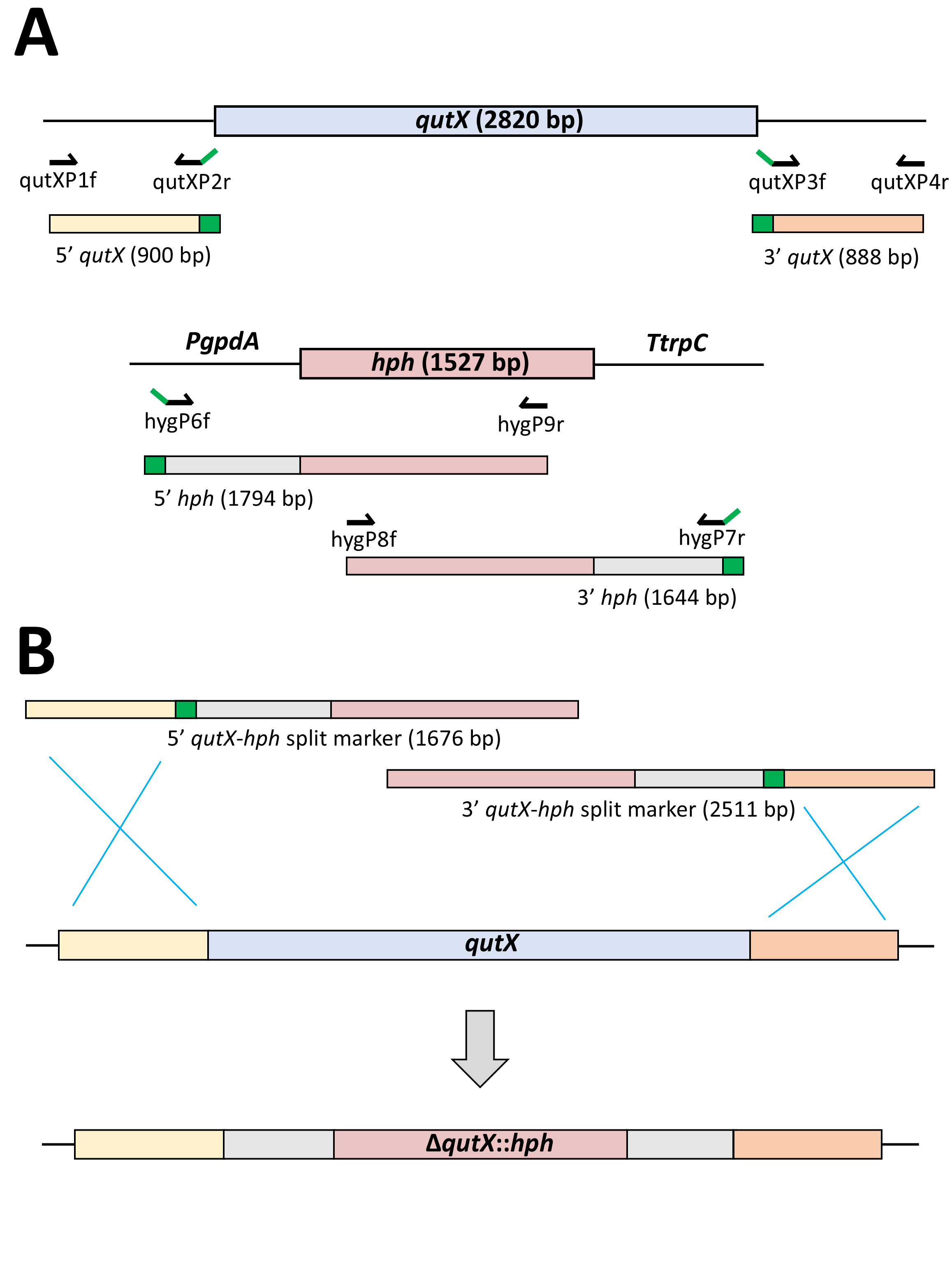

Supplement: jkaf199_Supplementary_Data [file jkaf199_supplementary_data.zip › Supplementary_Figure_1_G3-2025-406129.tif]

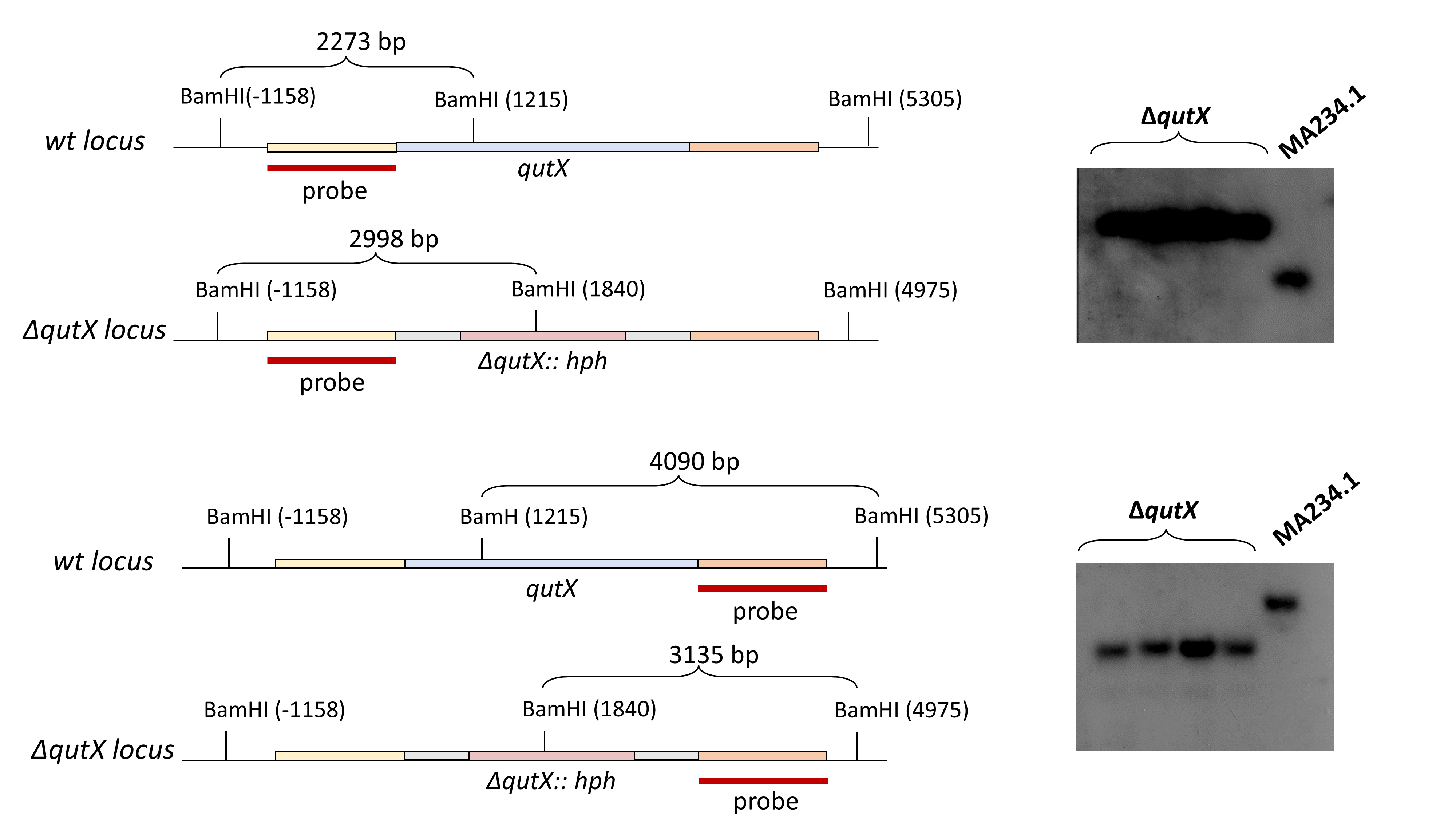

Supplement: jkaf199_Supplementary_Data [file jkaf199_supplementary_data.zip › Supplementary_Figure_2_G3-2025-406129.tif]

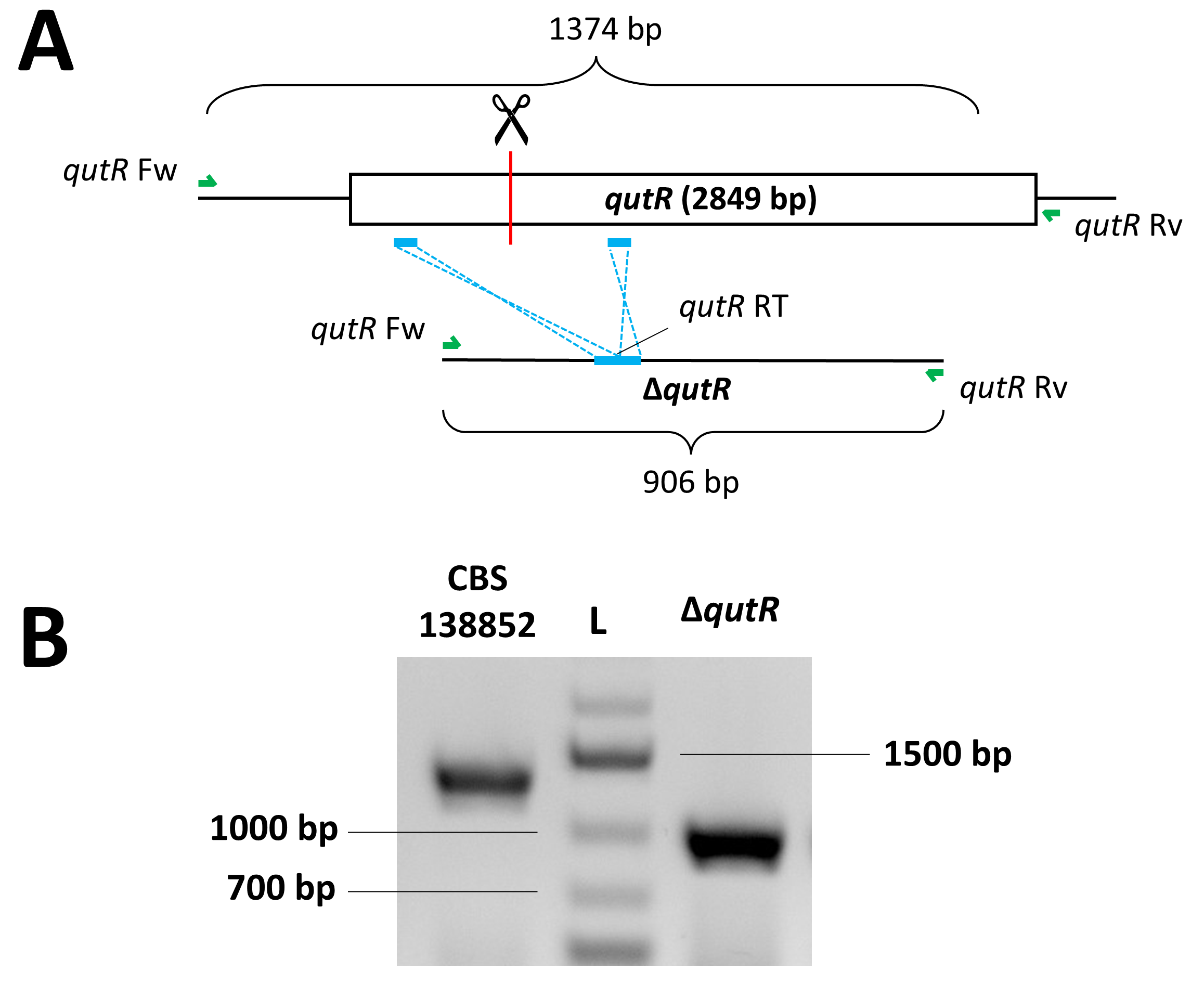

Supplement: jkaf199_Supplementary_Data [file jkaf199_supplementary_data.zip › Supplementary_Figure_3_G3-2025-406129.tif]

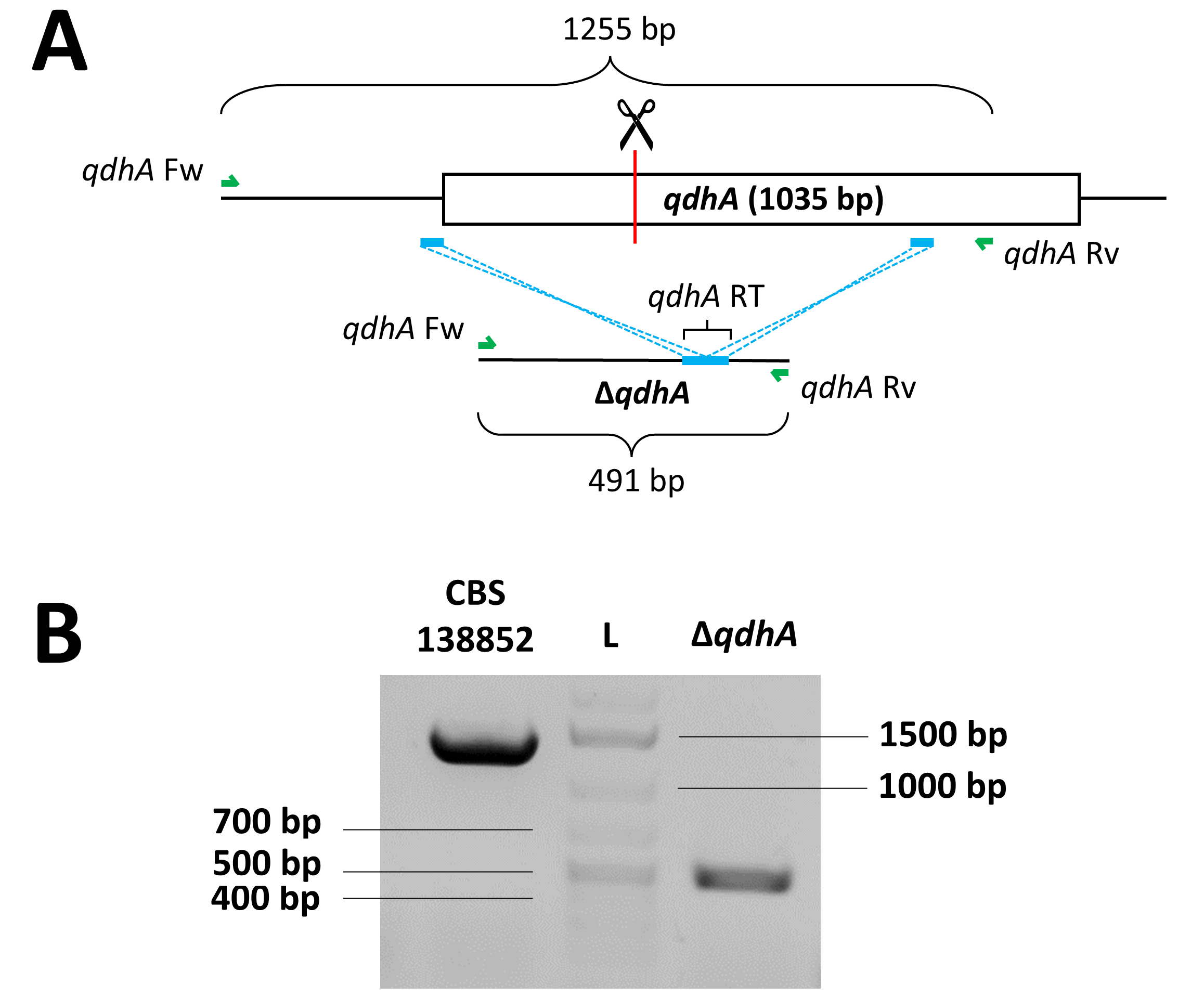

Supplement: jkaf199_Supplementary_Data [file jkaf199_supplementary_data.zip › Supplementary_Figure_4_G3-2025-406129.tif]

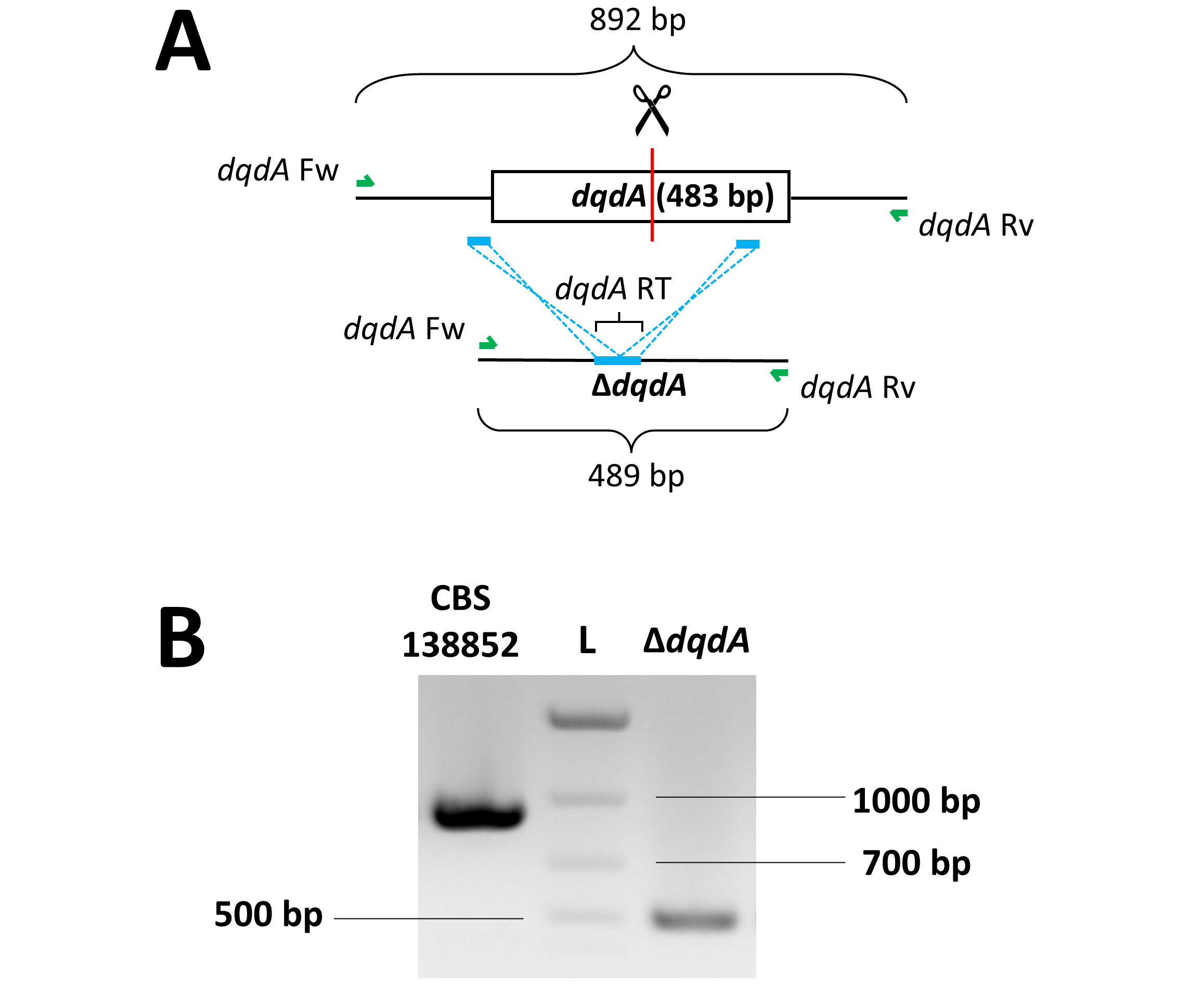

Supplement: jkaf199_Supplementary_Data [file jkaf199_supplementary_data.zip › Supplementary_Figure_5_G3-2025-406129.tif]

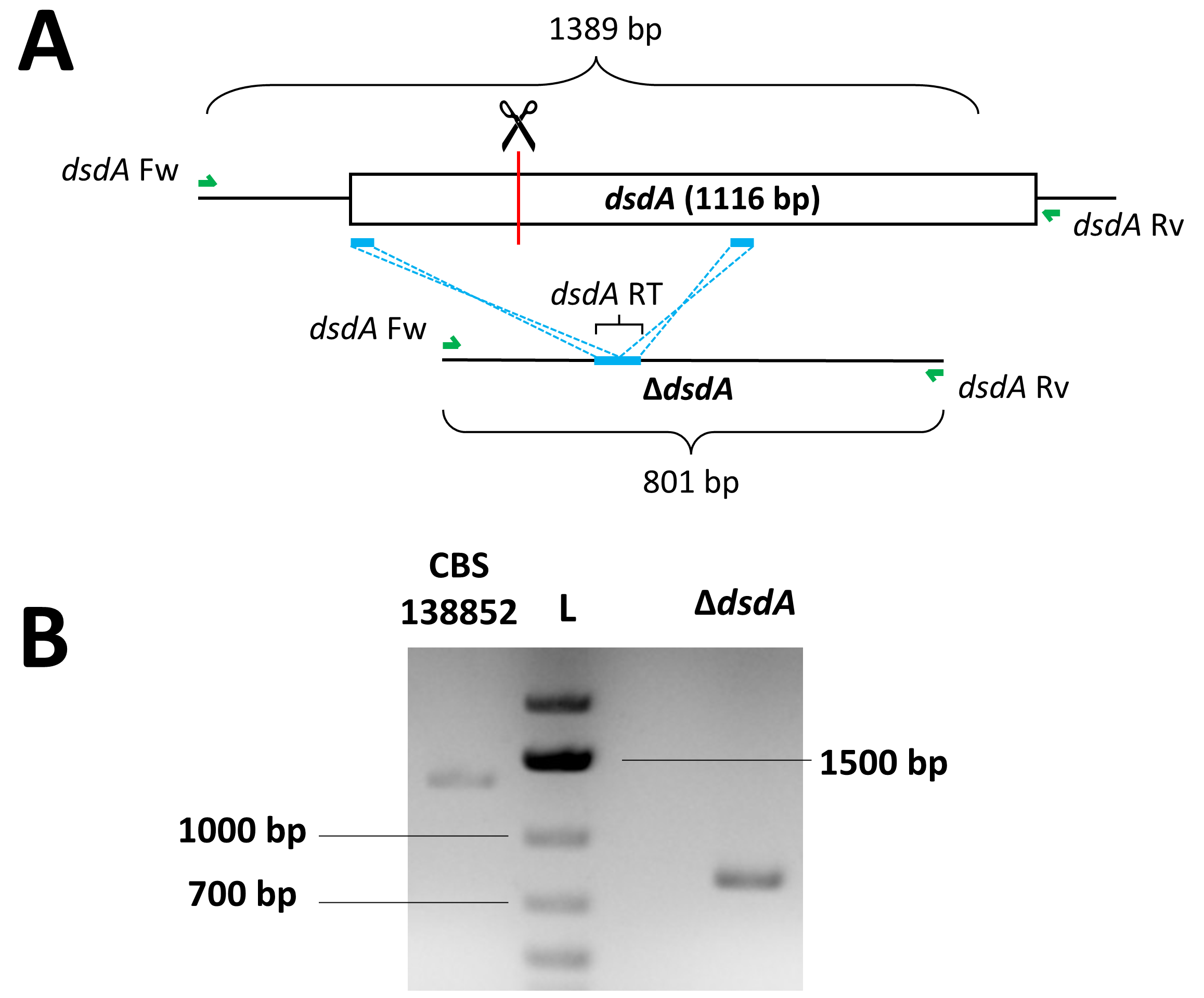

Supplement: jkaf199_Supplementary_Data [file jkaf199_supplementary_data.zip › Supplementary_Figure_6_G3-2025-406129.tif]

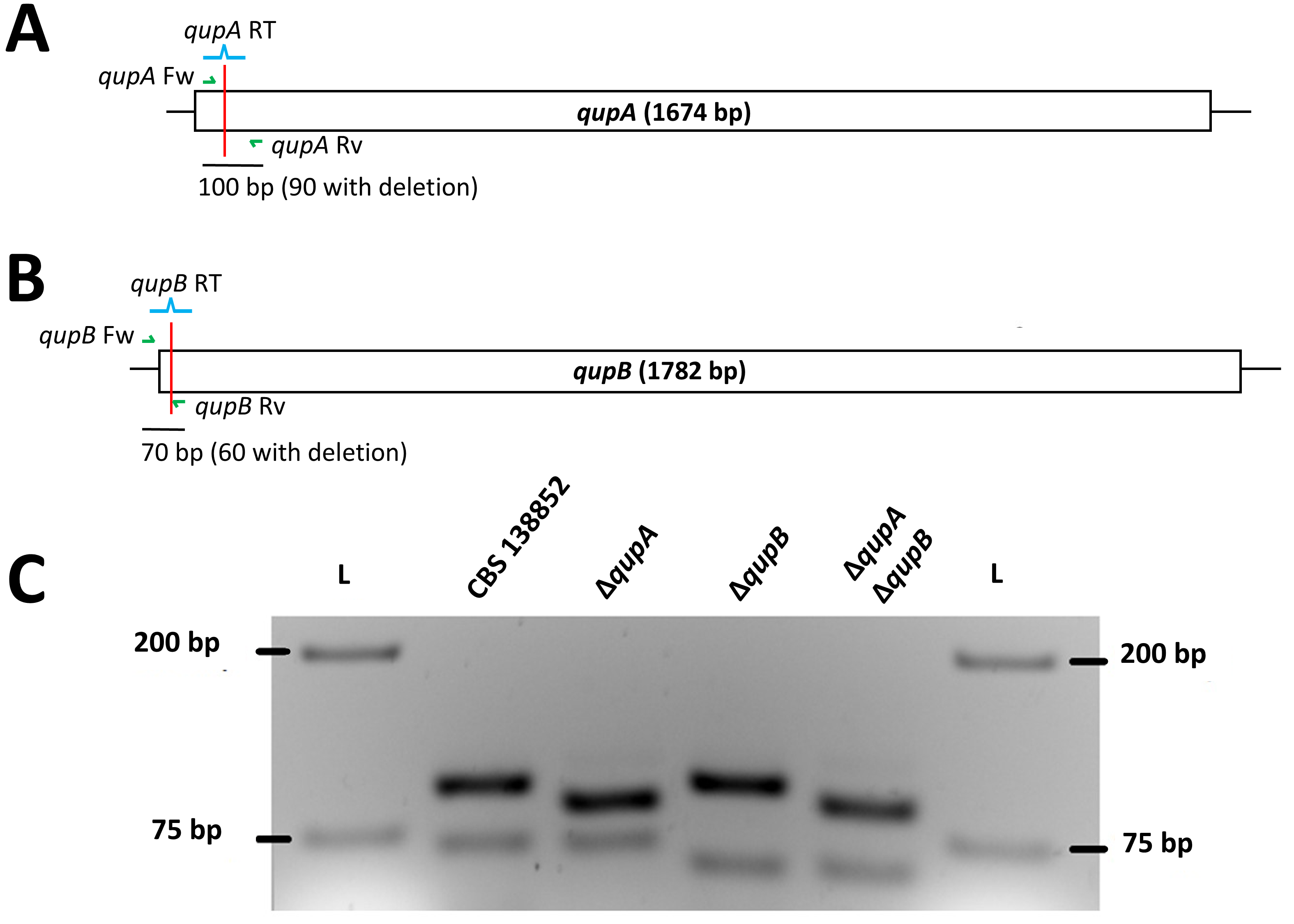

Supplement: jkaf199_Supplementary_Data [file jkaf199_supplementary_data.zip › Supplementary_Figure_7_G3-2025-406129.tif]

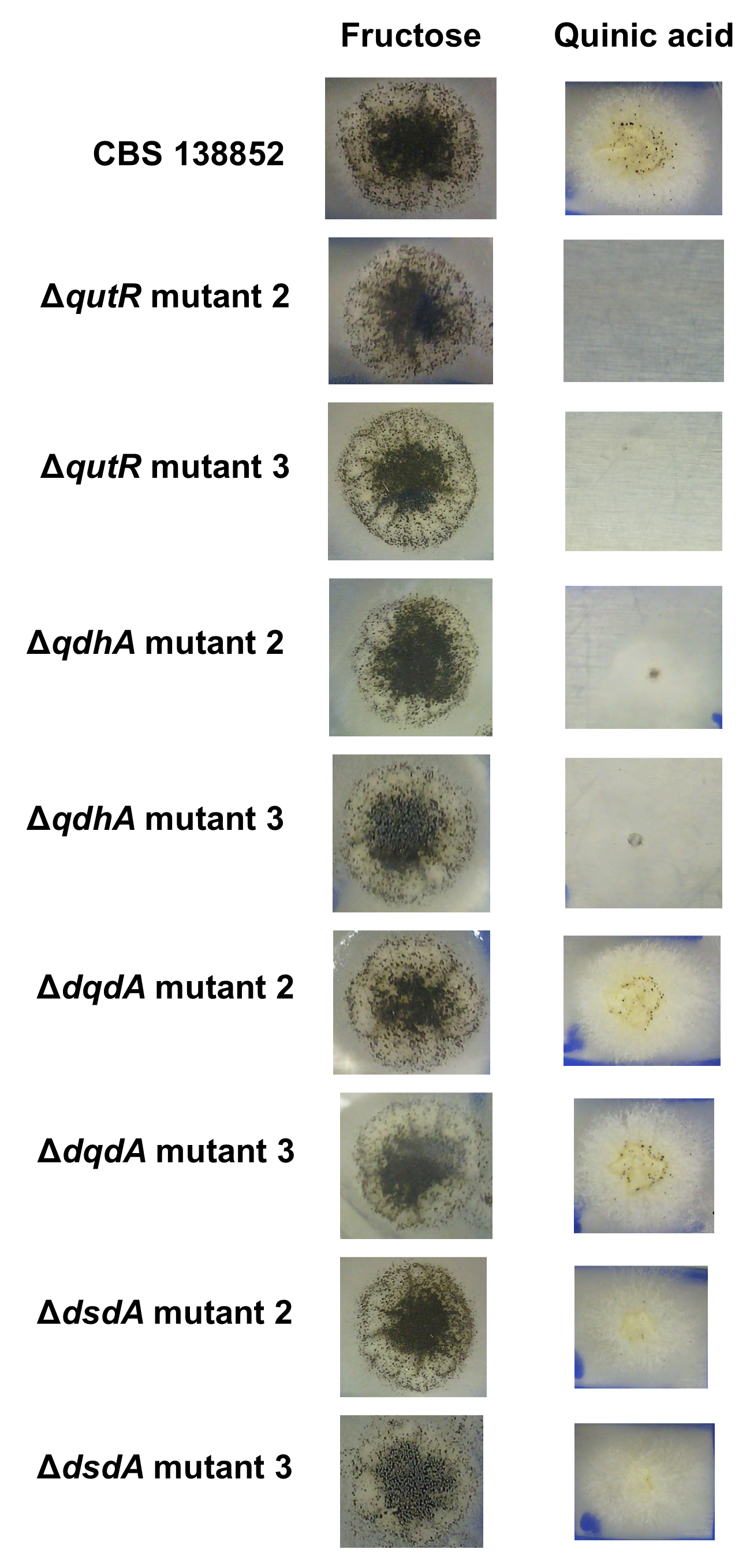

Supplement: jkaf199_Supplementary_Data [file jkaf199_supplementary_data.zip › Supplementary_Figure_8_G3-2025-406129.tif]

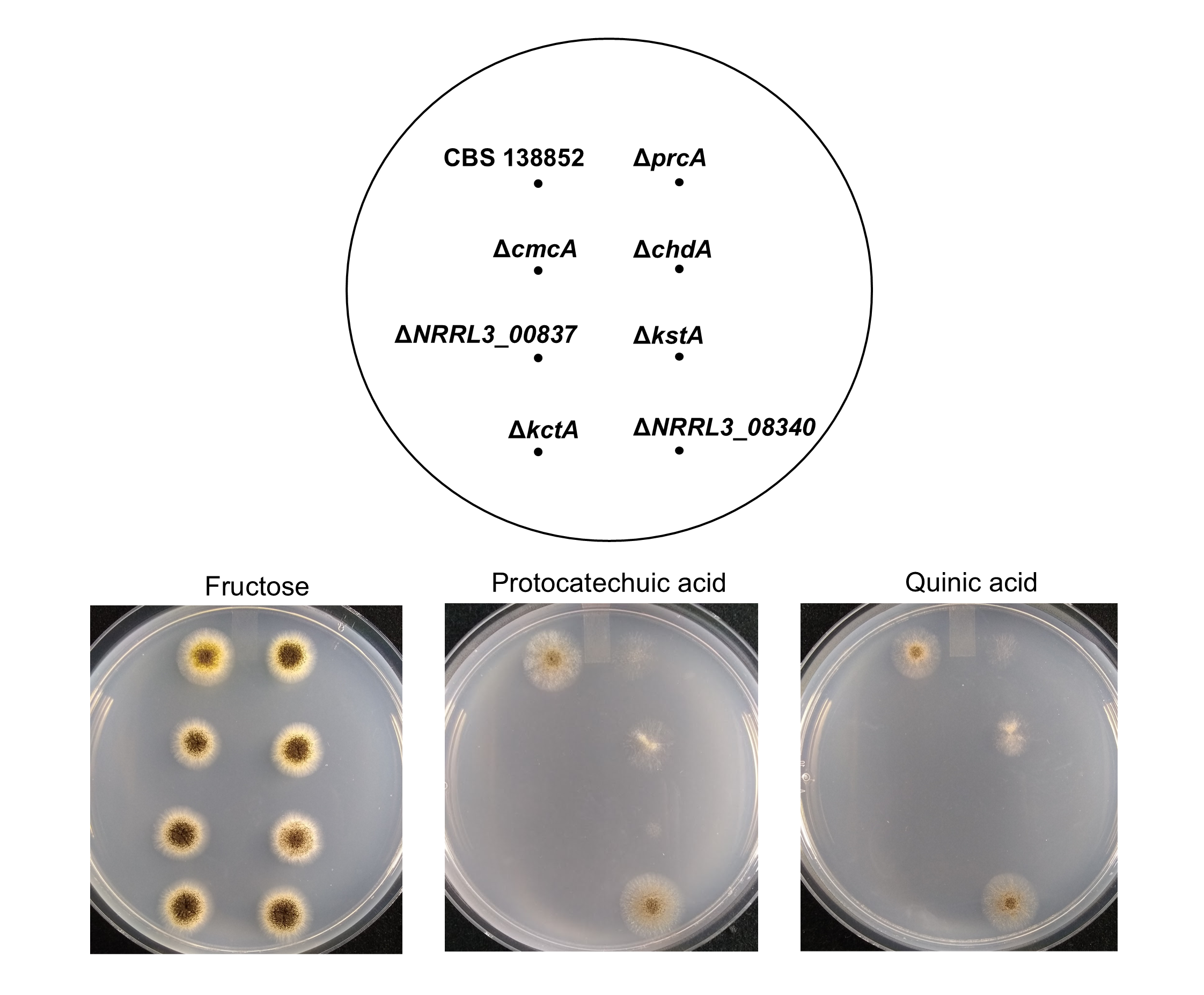

Supplement: jkaf199_Supplementary_Data [file jkaf199_supplementary_data.zip › Supplementary_Figure_9_G3-2025-406129.tif]
